# Cardiac device implantation in Fabry disease: A retrospective monocentric study

**DOI:** 10.1097/MD.0000000000004996

**Published:** 2016-10-07

**Authors:** Thomas Sené, Olivier Lidove, Joel Sebbah, Jean-Marc Darondel, Hervé Picard, Laurent Aaron, Olivier Fain, Thierry Zenone, Dominique Joly, Philippe Charron, Jean-Marc Ziza

**Affiliations:** a Department of Internal Medicine and Rheumatology, Reference Center for Lysosomal Storage Disorders (CRML, site Avron), Groupe Hospitalier Diaconesses Croix-Saint-Simon; b Inserm UMRS 974, Université Pierre & Marie Curie; c Department of Cardiology, Institut Mutualiste Montsouris; d Department of Clinical Research, Fondation Ophtalmologique Rothschild, Paris; e Department of Internal Medicine, Centre Hospitalier Jacques Coeur, Bourges; f Department of Internal Medicine, Hôpital Saint-Antoine, AP-HP, Université Pierre & Marie Curie, Paris; g Department of Internal Medicine, Centre Hospitalier de Valence, Valence; h Department of Nephrology, Hôpital Necker, AP-HP, Université René Descartes, Paris; i Referral Center For Cardiac Hereditary Diseases, Hôpital Pitié-Salpêtrière, AP-HP, Université Versailles-Saint-Quentin, Saint-Quentin-en-Yvelines, France.

**Keywords:** arrhythmia, cardiac device, conduction abnormalities, enzyme replacement therapy, Fabry disease (OMIM 301500), internal cardioverter-defibrillator, pacemaker

## Abstract

The incidence and predictive factors of arrhythmias and/or conduction abnormalities (ACAs) requiring cardiac device (CD) implantation are poorly characterized in Fabry disease (FD). The aim of our retrospective study was to determine the prevalence, incidence, and factors associated with ACA requiring CD implantation in a monocentric cohort of patients with confirmed FD who were followed up in a department of internal medicine and reference center for FD.

Forty-nine patients (20M, 29F) were included. Nine patients (4M, 5F; 18%) had at least one episode of ACA leading to device therapy. Six patients (4M/2F) required a pacemaker (PM) for sinus node dysfunction (n = 4) or atrioventricular disease (n = 2). One female patient required an internal cardioverter-defibrillator (ICD) to prevent sudden cardiac death because of nonsustained ventricular tachycardia (nSVT). One female patient required PM-ICD for sinus node dysfunction and nSVT. One patient underwent CD implantation before the diagnosis of FD. The annual rate of CD implantation was estimated at 1.90 per 100 person years. On univariate analysis at the end of the follow-up period, the factors associated with ACAs requiring CD implantation were as follows: delayed diagnosis of FD, delayed initiation of enzyme replacement therapy, age at the last follow-up visit, and severe multiorgan phenotype (hypertrophic cardiomyopathy, chronic kidney disease, and/or sensorineural hearing loss). On multivariate analysis, age at diagnosis of FD and age at the last follow-up visit were independently associated with an increased risk of ACAs requiring CD (*P* < 0.05).

Considering the high frequency of ACAs requiring CD implantation and the risk of sudden death in patients with FD, regular monitoring is mandatory, especially in patients with a late diagnosis of FD and/or with a severe phenotype. Regular Holter ECGs, therapeutic education of patients, and deliverance of an emergency card including a phenotype summary are crucial in the care of FD patients.

Available guidelines for device therapy and the efficacy of enzyme replacement therapy for arrhythmias or conduction abnormalities are discussed.

**Key points**

- ACAs requiring CD implantation represent a frequent complication in FD, with a prevalence of 18% and an annual incidence of 1.90% in our monocentric cohort.
- Patients with a delayed diagnosis of FD, severe phenotype (SHL, LVH, and/or CKD), and/or delayed initiation of ERT are at a high risk of ACAs requiring CDs.
- In this setting, close cardiac follow-up is mandatory to prevent these events.

## 1 Introduction

Fabry disease (FD) is an X-linked lysosomal storage disorder in which mutations of the *GLA* gene cause a deficiency in α-galactosidase A (OMIM 301500). Progressive lysosomal accumulation of its principal substrate, the neutral glycosphingolipid globotriaosylceramide (Gb3), and a subsequent fibrotic process cause a multisystemic vasculopathy that leads to a wide spectrum of manifestations. Both hemizygous males and heterozygous females can be affected, although male patients usually develop a more severe form of the disease, with a younger age at onset, than female patients.^[1]^ Life expectancy is reduced both in male (58.2 years) and in female (75.4 years) patients with FD.^[2]^

Cardiac involvement is one of the major causes of disability in FD. The cardiac phenotype of FD includes left ventricular hypertrophy (LVH), cardiac failure, and coronaropathy.^[3]^ Myocardial replacement fibrosis is a key feature of more advanced Fabry cardiomyopathy.^[4]^

Enzyme replacement therapy (ERT) with intravenous infusions of recombinant human alpha-galactosidase A consistently clears lysosomal inclusions from vascular endothelial cells.^[5]^ However, fibrosis in affected organs is not responsive to ERT, suggesting that treatment must be initiated early in the course of the disease to be optimally effective.^[1,6]^

Arrhythmia and/or conduction abnormalities (ACAs) have been reported and can cause significant morbidity and mortality in FD^[7–9]^; however, the incidence and predictive factors of ACAs requiring cardiac device (CD) implantation are poorly characterized in FD.

The aim of our study was to determine the prevalence and incidence of ACAs requiring CD implantation in FD and to characterize the factors associated with these events.

## 2 Methods

### 2.1 Study design

An observational, longitudinal, retrospective cohort design was used. The study conforms to the principles of the Helsinki Declaration. All patients provided informed consent before CD implantation.

### 2.2 Study population

The cohort consisted entirely of patients with FD who were consecutively evaluated at the Reference Center for Lysosomal Storage Disorders (CRML) from 1st November 2001 until 1st January 2016. The patients were followed as in- and outpatients at Bichat University Hospital (Paris, France) from 1st November 2001 until 1st March 2012 and at Groupe Hospitalier Diaconesses Croix-Saint-Simon Hospital (Paris, France) from 1st March 2012 until 1st January 2016. The diagnosis of FD was determined by measuring leukocyte alpha-galactosidase A enzyme activity and by identifying mutations in the *GLA* gene. The patients followed in our institution were all adults (adulthood defined in French hospitals as age > 15 years and 3 months). Decision of initiation or cessation of ERT in FD patients was consistent with the French Recommendations (Protocole National de Diagnostic et de Soins – Maladie de Fabry – Haute Autorité de Santé – www.has-sante.fr).

### 2.3 Follow-up and endpoint

A clinical review was performed every 6–12 months (or earlier if there was a clinical event) until 1st January 2016. A full-blown phenotype of FD was assessed, including an early phenotype (pain in the extremities, heat intolerance, angiokeratoma, corneal dystrophy). Routine investigations comprised creatinine levels and daily proteinuria testing, audiometry, electrocardiography, echocardiography, and brain magnetic resonance imaging (MRI).

The main endpoint was CD implantation with a pacemaker (PM) or internal cardioverter-defibrillator (ICD). The indications for CD implantation were determined by reviewing clinical charts.

### 2.4 Definitions

The follow-up period was calculated from the date of diagnosis of FD to the most recent clinical evaluation.

Cardiac involvement was defined as the presence of hypertrophic cardiomyopathy and/or ACAs requiring CD implantation. LVH was defined as posterior wall thickness in diastole and/or septal wall thickness in diastole > 13 mm. Hypertrophic cardiomyopathy was defined according to the usual recommendations.^[1,10]^

Renal involvement was defined as chronic kidney disease (CKD) and/or proteinuria higher than 0.3 g/day. CKD was defined as an estimated glomerular filtration rate (eGFR) lower than 60 mL/min/1.73 m^2^, and eGFR was evaluated using the Modification of Diet in Renal Disease Study (MDRD) estimation.

Neurological involvement was defined as transient ischemic attacks or stroke.

Sensorineural hearing loss (SHL) was defined as a hearing threshold above the 95th percentile for age- and sex-matched normal controls.

### 2.5 Statistical analysis

Continuous variables were summarized by their medians, interquartile range (IQR), and extreme values. Categorical variables were described by the counts of each modality, the corresponding percentage being shown in parentheses. Statistical comparisons of continuous variables were made using unpaired Student *t*-test (if normality assumption could be sustained) or Wilcoxon exact test (otherwise). Statistical comparisons of binary variables were made using χ^2^ test. The results were considered significant for a *P*-value < 0.05.

The annual event rate was computed by dividing the number of patients reaching the endpoint by the total follow-up period (calculated from the date of diagnosis of FD to the time of reaching the endpoint, or, if not reached, to the most recent clinical evaluation).

Multivariate analysis was conducted on key predictive factors using stepwise descended variable selections. All analyses were performed using the free software R statistical package (version 3.1.1; 2014–07–10).

## 3 Results

Forty-nine patients (female: 29; male: 20) from 26 families (1 to 8 patients per family) were evaluated during the study period.

### 3.1 Patient population and clinical characteristics

**Figure 1:**
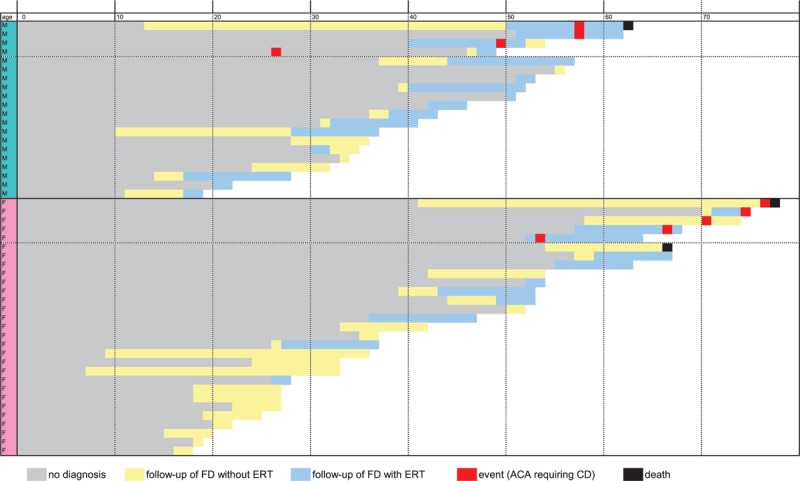
Overview of the main characteristics of 49 patients with FD followed up in the cohort. ACA = arrhythmia and/or conduction abnormality, CD = cardiac device, ERT = enzyme replacement therapy, FD = Fabry disease.

Table 1 summarizes the main characteristics (stratified by sex) of the patients at the end of the follow-up period. Figure 1 illustrates the main characteristics for each patient during the follow-up.

**Table 1:**
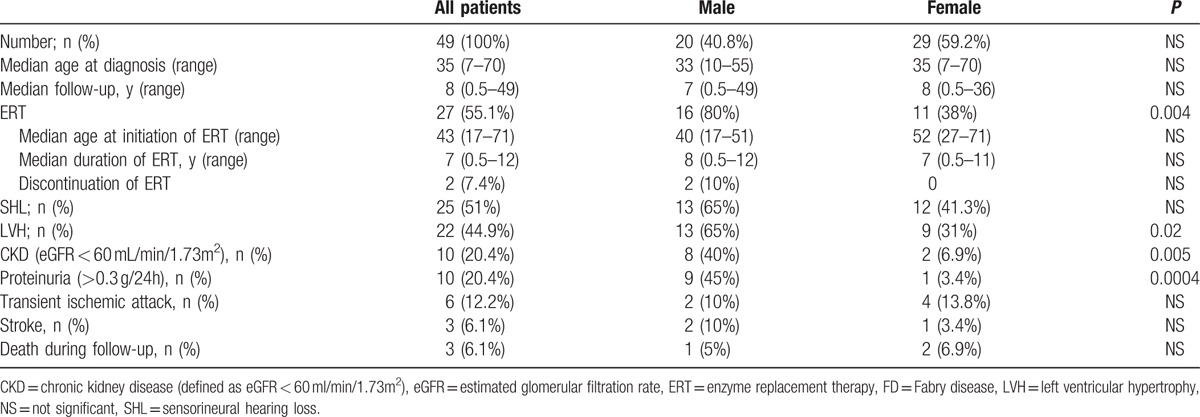
Characteristics of patients with FD at the end of the follow-up period.

The median age at diagnosis of FD was 35 (IQR 20–50, range 7–70). The median follow-up since diagnosis of FD was 8 years (IQR 2–11 years, range 6 months to 49 years).

Twenty-seven patients (55.1%; female: 11, male: 16) received ERT. Male patients were more likely to be treated with ERT (*P* = 0.004). The median age at initiation of ERT was 43 (IQR 31–51 years, range 17–71 years). The median duration of ERT was 7 years (IQR 1–10, range 0.5–12). Two male patients discontinued ERT during follow-up, one for an anaphylactoid reaction and one for nonobservance.

Twenty-five patients (51%) had SHL, 22 (45%) had LVH, 10 (20%) had CKD, and 9 (18%) had a transient ischemic attack and/or stroke. The prevalences of LVH, CKD, and proteinuria were significantly higher in male patients.

Three patients (6%) died during follow-up at ages of 61 (male, septic shock), 65 (female, pancreatic cancer), and 76 (female, massive pulmonary embolism).

**Figure 2:**
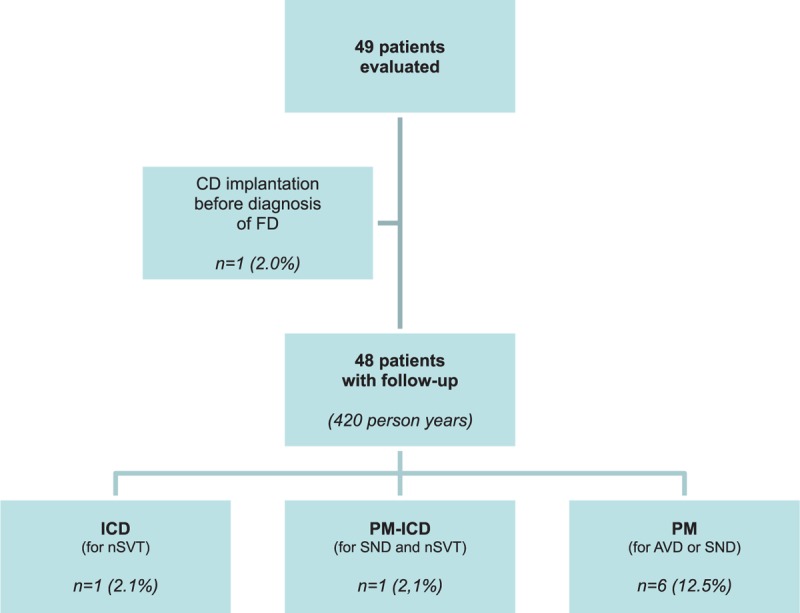
Study cohort and outcome. AVD = atrioventricular disease, CD = cardiac device, FD = Fabry disease, ICD = internal cardioverter-defibrillator, nSVT = nonsustained ventricular tachycardia, PM = pacemaker, SND = sinus node disease.

Nine patients (18%; female: 5, male: 4) with FD had ACAs requiring CD. One patient underwent CD implantation before the diagnosis of FD (Fig. 2).

### 3.2 Description of ACAs requiring CD implantation

Table 2 summarizes the type of ACA, characteristics of the patients at CD implantation, and outcome.

**Table 2:**
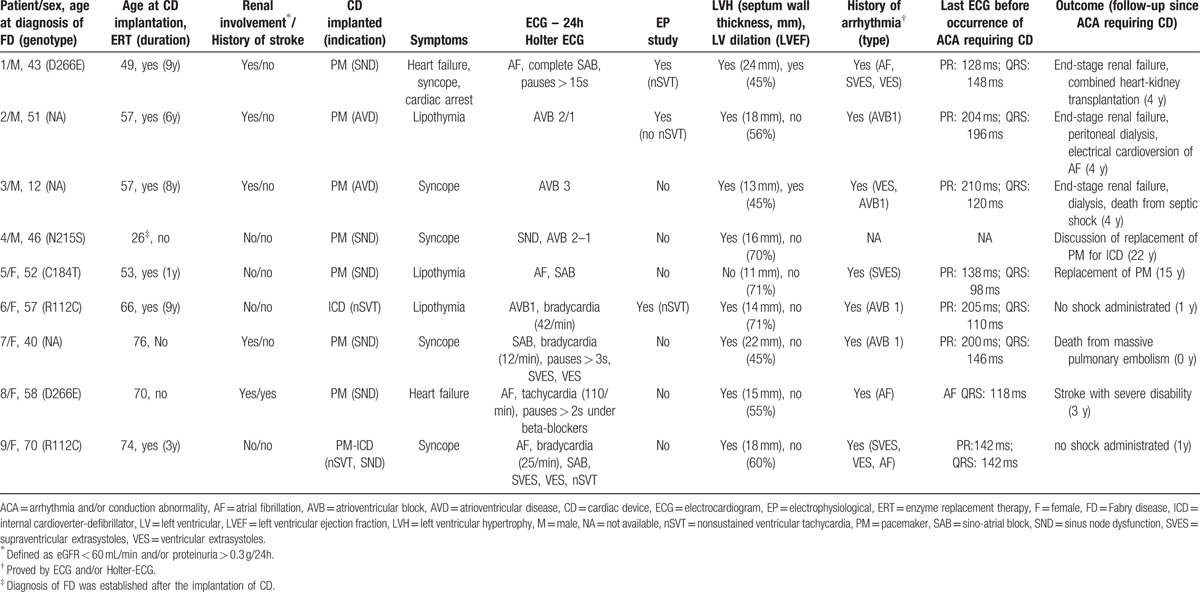
Characteristics of FD patients with ACA requiring CD implantation.

The median age at CD implantation was 57 (IQR 53–70, range 26–76). All but 1 patient (patient 4; Table 2) was older than 45 at the time of CD implantation. The age at CD implantation tended to be older in females (70, IQR 66–74, range: 53–76) than in males (53, IQR 43–57, range 26–57) (*P* = 0.07). Six patients (4M/2F) required PM for sinus node dysfunction (n = 4) or atrioventricular disease (n = 2). One female patient suffering from lipothymia required an ICD to prevent sudden cardiac death because of nonsustained ventricular tachycardia (nSVT). One female patient with syncope required PM-ICD for sinus node dysfunction and nSVT. Eight of 9 patients had a history of ACAs, as demonstrated by electrocardiogram (ECG) and/or Holter monitoring before CD implantation. Seven of 9 patients had QRS duration ≥110 ms before the occurrence of ACA requiring CD. In these 9 patients, the median septal wall thickness was 16 mm (range 11–24), and 8 patients had LVH. Two patients had left ventricular dilation. Three patients had left ventricular ejection fractions (LVEF) below 50%. Five patients had renal involvement, and 1 patient had a history of stroke.

Six of these 9 patients received ERT for a median duration of 7 years (range 1–8). One patient (patient 4; Table 2) did not receive ERT because FD diagnosis was not made at the time of ACA requiring CD. One patient (patient 7; Table 2) declined ERT despite heart and kidney involvement of FD. The third untreated patient (patient 8; Table 2) had a severe multiorgan involvement, with an unfavorable benefit/risk balance.

The median follow-up since CD implantation was 4 years (IQR 1–4, range 0–22). Three patients developed terminal renal failure. One patient underwent combined heart–kidney transplantation. One patient had a stroke resulting in severe disability. Two patients died: a 76-year-old female early after CD implantation from a massive pulmonary embolism and a 61-year-old male from septic shock 4 years after CD implantation. PM replacement for an ICD is under discussion in one case (patient 4; Table 2).

### 3.3 Factors associated with CD implantation

Table 3 summarizes the main characteristics of the patients (stratified by occurrence of ACAs requiring CD implantation) at the end of the follow-up period.

**Table 3:**
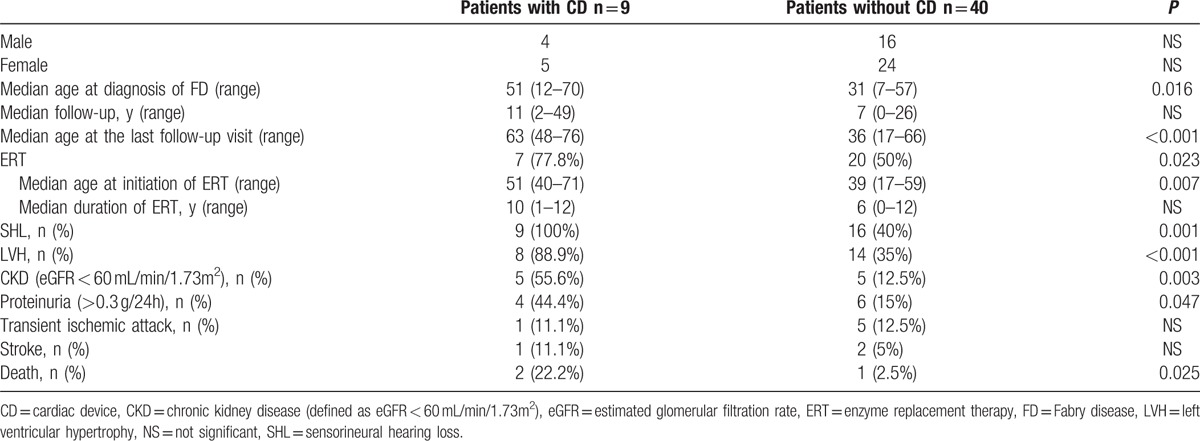
Characteristics of FD patients with and without CD at the end of the follow-up period.

In univariate analysis, patients with CDs were older at the last follow-up visit (*P* < 0.001), had a delayed diagnosis of FD (*P* = 0.016), were more likely to be treated with ERT during follow-up (*P* = 0.023), were significantly older at the initiation of ERT (*P* = 0.007), and had a higher frequency of SHL (*P* = 0.001), LVH (*P* < 0.001), and CKD (*P* = 0.003) than patients without CDs. In multivariate analysis, age at FD diagnosis (*P* = 0.024) and age at the last follow-up visit (*P* = 0.025) were independently associated with a higher risk of ACAs requiring CD implantation [OR for each additional year: 1.19 (IC: 1.06–1.47) and 1.22 (IC: 1.06–1.56), respectively].

### 3.4 Incidence of CD implantation since diagnosis of FD

The study cohort and outcomes are shown in Fig. 2. Forty-eight patients (without a CD at diagnosis of FD) were followed-up for 420 patient years following diagnosis of FD. At the end of the study, 8 patients (16.7%) received a CD. The annual implantation rate for any CD was estimated at 1.90 per 100 person years (CI: 1.40–2.26).

## 4 Discussion

In this retrospective study, we determined that an ACA requiring device therapy represents a frequent complication in FD, with a prevalence of 18% and an annual incidence following the diagnosis of FD of 1.90%. A delayed diagnosis of FD, severe phenotype (SHL, LVH/and or CKD), and/or delayed initiation of ERT are factors associated with CD implantation. Notably, female patients may also need CDs (at an older age than males), and ACAs requiring CD implantation may occur precociously, even before the diagnosis of FD. Occurrence of ACAs leading to device therapy was associated with significant morbidity and mortality from the cardiac as well as the global phenotype associated with these events.

### 4.1 Epidemiology of CD implantation in FD

In a retrospective longitudinal study of 207 patients with FD, the prevalence of PM implantation was 8.7%, and the annual incidence was 1.07 per 100 person years.^[2,11]^ This rate was estimated to be > 25 times higher than that observed in the general population. Our study confirms, with an annual incidence of 1.90 per 100 person years, the high frequency of ACAs requiring CD implantation compared with general population.^[12]^ Notably, in our study, the annual event rate of CD implantation was calculated from the diagnosis of FD to the last follow-up visit and included ICD implantation for prevention of nSVT.

Krämer et al also reported that patients who have significant fibrosis on MRI and those who have nSVT on Holter monitor are at higher risk for rhythmic complications.^[5,13]^

Patel et al^[11]^ determined in a univariate analysis that age at first evaluation, male sex, Mainz Severity Score Index (MSSI) (used to assess the overall severity of FD), indexed left ventricular (LV) mass, long PR interval, and QRS duration were independent predictors of PM implantation. In a multivariate analysis, age and QRS duration remained the only significant predictors of device placement. The presence of a cardiac variant was not protective.^[1,6,11]^

In our study, we identified new factors associated with CD implantation: severe phenotype (SHL, LVH/and or CKD), delayed diagnosis of FD, and delayed initiation of ERT. A full-blown phenotype of FD including SHL, LVH, and/or CKD was studied rather than the MSSI because the MSSI does not include SHL and can diminish when a patient is organ-transplanted (2 patients in our cohort). In our study, which included more female than male FD patients, we did not find differences between sexes in CD implantation. Our data confirm that females with FD are not only carriers but are also often severely affected, particularly after 50 years of age.

### 4.2 FD as a vascular, profibrotic, and multisystemic disease

As illustrated by a regular absence of liver and spleen enlargement, FD should not be considered only a storage disorder. The main cell involved in the pathophysiology of FD is the endothelial cell. In a cluster analysis, Kaminsky et al found that SHL, CKD, LVH, and stroke were strongly associated with the disease, illustrating that FD should be considered mainly a vascular disease.^[3,14]^

The development of fibrosis is a common and major feature in FD in different organs, such as the kidney (glomerulosclerosis) and heart.^[4,7–9,15]^ End-stage renal disease was also found to be the strongest indicator of cardiovascular disease progression in FD.^[15]^ This emphasizes that organ involvement in FD is not independent but is linked to ischemia and fibrosis.

### 4.3 Influence of ERT on occurrence of ACAs requiring CD implantation

ERT has been found to clear Gb3 from tissues, improve cardiovascular conduction, reduce LV mass, decrease diastolic dysfunction, and improve systolic function^[6]^; however, to our knowledge, ERT has not been found to prevent ACAs requiring CD.

Development of fibrosis cannot be stopped or reversed by ERT.^[13]^ Schmied et al recently described that an abnormal ECG at the time of treatment initiation is significantly associated with cardiac disease progression. These authors also found that maximal treatment benefit was found when ERT was initiated before ECG abnormalities develop.^[16]^ These findings suggest that treatment must be initiated early in the course of the disease to be optimally effective and that early diagnosis of FD is crucial to avoid the “point of no-return” because of irremediable fibrosis.^[1,6]^ As illustrated in Fig. 1, most of our patients with ACAs requiring CDs had a delayed diagnosis of FD and then delayed initiation of ERT, with probable irreversible fibrosis.

### 4.4 Device therapy in FD

To date, there are no randomized trials or validated prediction models to guide CD implantation in FD patients. Table 4 summarizes the European Society of Cardiology (ESC) Guidelines of CD implantation in Hypertrophic Cardiomyopathy, which include FD.^[17]^ The ESC guidelines for hypertrophic cardiomyopathy have developed a sudden cardiac death (SCD) risk score to guide ICD implantation but specifically state that this score should not be used for infiltrative diseases, such as FD.^[17]^

**Table 4:**
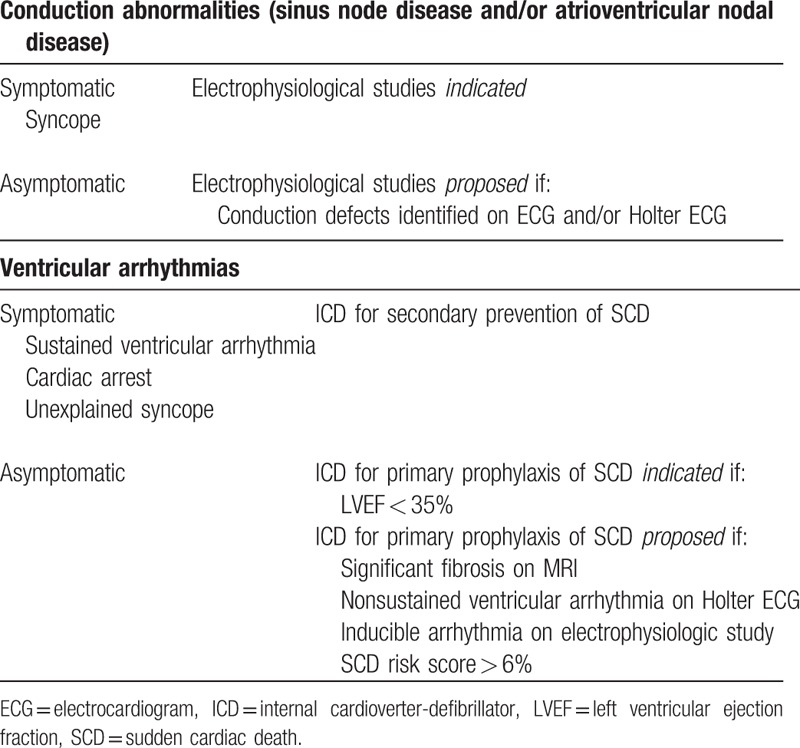
Proposed guidelines for cardiac device implantation in FD patients, adapted form ESC Guidelines for Hypertrophic Cardiomyopathy.

At least yearly ECGs, 24-hour Holter monitoring, and echocardiography are recommended in FD patients, even those who are asymptomatic, to detect patients at high risk and to guide CD implantation. New preventive tools (such as > 7 days Holter ECG or loop recorder implantation) proposed in sarcomeric hypertrophic cardiomyopathies require further research in FD.

### 4.5 Limitations

Our study included only 49 patients. Nevertheless, FD is a rare disease, and this monocentric cohort of FD patients followed up in a department of internal medicine is illustrative of the phenotypic heterogeneity of FD. Such a limited number of statistical units did not allow to build robust multivariate models with many simultaneous predictors; therefore, it is likely that such approach was not powerful enough to elicit all important factors associated with ACAs requiring CD, for instance LVH, CKD, and/or SHL.

Due to the retrospective design and the lack of several items of data, the clinical, radiological (cardiac MRI), and genetic predictors of rhythmic events requiring CDs were not determined. Moreover, the impact of ERT on the occurrence of CD implantation was difficult to assess because of the short ERT duration in adult and often elderly patients.

## 5 Conclusion

FD patients are at high risk for arrhythmia, conduction abnormality, and/or sudden cardiac death. A close cardiac follow-up seems mandatory in FD patients, especially in cases of delayed diagnosis of FD, severe multiorgan phenotype, and/or LVH.

Routine follow-up testing in patients with FD should include at least a yearly clinical review (palpitations, syncope, familial history of malignant arrhythmias, and cardiac death), electrocardiogram, 24-hour Holter ECG, and echocardiogram. We also strongly support that FD patients should always keep an emergency card with them and a reference ECG.

Long-term follow-up studies of FD patients under ERT or emerging alternative therapies (such as chaperon molecules or substrate reduction therapies) will determine the abilities of these treatments to prevent ACAs requiring CDs.
